# Bidirectional regulatory effects of Cordyceps on arrhythmia: Clinical evaluations and network pharmacology

**DOI:** 10.3389/fphar.2022.948173

**Published:** 2022-08-19

**Authors:** Lijuan Wang, Helin Sun, Meina Yang, Yulin Xu, Linlin Hou, Haomiao Yu, Xueyin Wang, Zhongwen Zhang, Jinxiang Han

**Affiliations:** ^1^ Department of Endocrinology and Metabolism, Affiliated Hospital of Weifang Medical University Weifang China, Shandong Provincial Qianfoshan Hospital & The First Affiliated Hospital of Shandong First Medical University, Neck-Shoulder and Lumbocrural Pain Hospital of Shandong First Medical University, Jinan, China; ^2^ Department of Endocrinology and Metabolism, The First Affiliated Hospital of Shandong First Medical University, Jinan, China; ^3^ NHC Key Laboratory of Biotechnology Drugs(Shandong Academy of Medical Sciences), Biomedical Sciences College, Shandong First Medical University, Jinan, China; ^4^ Key Laboratory of Biotechnology Drug (Shandong Academy of Medical Sciences), Biomedical Sciences College and Shandong Medicinal Biotechnology Centre, Shandong First Medical University and Shandong Academy of Medical Sciences, Jinan, China; ^5^ Ambulatory Surgery Centers, Tai’an City Central Hospital, Tai’an, China; ^6^ Department of Endocrinology and Metabolism, Affiliated Hospital of Weifang Medical University, Weifang, China

**Keywords:** cordyceps, traditional Chinese medicine, arrhythmia, network pharmacology, meta-analysis

## Abstract

**Background:** Cordyceps is a precious Chinese herbal medicine with rich bio-active ingredients and is used for regulating arrhythmia alongside routine treatments. However, the efficacy and potential mechanisms of Cordyceps on patients with arrhythmia remain unclear.

**Methods:** Randomized controlled trials of bradycardia treatment with Cordyceps were retrieved from diverse databases and available data. Dichotomous variables were expressed as a risk ratio (RR) with a 95% confidence interval (CI). Continuous variables were expressed as a standardized mean difference (SMD) with a 95% CI. Network pharmacology was used to identify potential targets of Cordyceps for arrhythmia. Metascape was used for gene ontology (GO) and genome (KEGG) pathway enrichment analysis.

**Results:** Nineteen trials included 1,805 patients with arrhythmia, of whom 918 were treated with Ningxinbao capsule plus routine drugs, and, as a control, 887 were treated with only routine drugs. Six trials reported on bradycardia and the other 13 on tachycardia. Treatment with Cordyceps significantly improved the total efficacy rate in both bradycardia (RR = 1.24; 95% CI, 1.15 to 1.35; P_z_ <0.00001) and tachycardia (RR = 1.27; 95% CI, 1.17 to 1.39; P_z_ <0.00001). Cordyceps also had beneficial secondary outcomes. No serious adverse events occurred in patients treated with Cordyceps. The results of KEGG pathway enrichment analysis were mainly connected to adrenergic signaling in cardiomyocytes and the PI3K-Akt signaling pathway. IL6, TNF, TP53, CASP3, CTNNB1, EGF, and NOS3 might be key targets for Cordyceps in the treatment of arrhythmia.

**Conclusion:** This study confirmed that Cordyceps has a certain positive effect on the treatment of arrhythmia and that its main mechanism may be through the regulation of adrenergic signaling in cardiomyocytes and the PI3K-Akt signaling pathway.

## 1 Introduction

Cardiovascular disease takes the lives of 17.7 million people every year, accounting for 31% of all global deaths, and this number will possibly rise to 23.6 million by 2030 (Borghi et al., 2018). Cardiac arrhythmias are mostly found in patients with organic cardiovascular diseases and are abnormalities or disturbances in the normal activation or beating of the heart muscles (Fu, 2015). Arrhythmias are a set of life-threatening cardiovascular diseases that can be divided into two types: bradyarrhythmias and tachyarrhythmias (Hategan et al., 2017; Black et al., 2019). Although the therapies for arrhythmias have attracted much attention, the efficacy and safety of modern medicines in the treatment of this disease are not satisfactory. Treatment with anti-arrhythmia drugs is not proven to reduce mortality, and the side effects of anti-arrhythmia drugs in patients’ digestive and cardiovascular systems have been brought to the forefront (Hao et al., 2015).

In recent years, with the development of evidence-based studies, the effects of traditional Chinese medicine (TCM) have been further confirmed and valued. Chinese herbs and herbal formulas have been used for arrhythmia since the 1970s (Hao et al., 2015; Hao et al., 2017). Herbal formulas can improve symptoms and adjust the heart rate to a normal level in patients with arrhythmia (Liu et al., 2018). Cordyceps, under the trade name “Ningxinbao capsule”—a liquid submerged fermentation of dry mycelium powder (Hao et al., 2017; Hategan et al., 2017)—is a standardized traditional Chinese medication that is widely used in the arrhythmia treatment, including for atrioventricular block, refractory bradycardia, and conduction block (Zhang et al., 2017; Das et al., 2020). However, there is no credible scientific evidence supporting the use of Cordyceps in treating and preventing arrhythmia. Currently available randomized controlled trials (RCTs) are limited to small sample sizes with inconsistent outcomes. Drawing authoritative conclusions about the genuine benefits and possible side effects is thus difficult. Network pharmacology has become a new approach to drug mechanism research and drug development. In recent years, a variety of related databases and tools have provided crucial support for TCM network pharmacology (Zhang et al., 2019). We thus performed this meta-analysis to estimate the effectiveness and safety of Cordyceps compared to contemporary anti-arrhythmia drugs and placebos, by assessing total effectiveness rates, improvement in symptoms, changes in heart rates, and the incidence of drug-related adverse effects. Network pharmacology was also used to identify the potential mechanisms of Cordyceps for treating arrhythmia.

## 2 Materials and methods

### 2.1 Meta-analysis

#### 2.1.1 RCT preparation

Literature was retrieved from the PubMed Database, the China National Knowledge Infrastructure (CNKI), the Wanfang Database, the China Scientific Journal Database (VIP), and the Chinese Biomedicine Literature Service System (SinoMed). Published review articles, editorials, and information from the internet (http://www.clinicaltrialresults.org, http://www.clinicaltrials.gov, and http://www.theheart.org), were also considered, covering the period from the earliest possible date up to December 30, 2021. Bradyarrhythmia, which is basically a heart rate below 60 beats per minute, can be due to sinus, atrial or junctional bradycardia, or problems with the conduction system, e.g., sinus bradycardia, sinoatrial block, atrioventricular block, and others (Kumar et al., 2012; Sidhu and Marine, 2020). Tachyarrhythmia, usually defined as a heart rate of more than 100 beats per minute, mainly involves premature beat, atrial flutter, atrial fibrillation, and so on (Boateng, 2013; Sagris et al., 2021). The search algorithms for PubMed were as follows: “Ningxinbao,” “Cordyceps,” AND (“arrhythmia” or “arrhythmia” or “bradyarrhythmia” or “bradycardia” or “sinoatrial block” or “sinus arrest” or “atrioventricular block” or “sick sinus syndrome” or “tachyarrhythmia” or “tachycardia” or “premature beat” or “premature ventricular contractions” or “tachycardia” or “flutter” or “fibrillation”), AND “randomized,” AND “blind,” with no restriction on subheadings. Similar but appropriate search terms were used for other literature databases or search engines. The reference lists of all included articles were searched for other relevant citations, and articles not included in the above electronic databases were checked manually. Whenever necessary, the authors of the included trials were contacted for additional information. Available citations were searched and selected by two authors (L. J. and H. L.) independently and in duplicate; two authors (L. J. and H. L.) abstracted data and assessed the methodological quality of the trials.

#### 2.1.2 Inclusion and exclusion criteria of the studies

The inclusion criteria were as follows: 1) Study subjects were diagnosed as arrhythmic according to the corresponding guidelines. 2) All patients were randomized to receive treatment with Ningxinbao and contemporary medications, or contemporary medications only. 3) The sample size in each study group was ≥10 cases. 4) Follow-up in each study group was ≥2 weeks. 5) Quantitative measurements of surrogate endpoints and/or adverse cardiovascular events and/or adverse drug effects were available to facilitate outcome analysis.

Exclusion criteria were as follows: 1) Studies were non-randomized or non-blinded. 2) Patients enrolled had no definite diagnosis. 3) Different TCM medications were compared. 4) Studies reported only symptomatic changes in patients without objective laboratory measurements. 5) Methodological quality was poor with a Jadad score of <2.

#### 2.1.3 Statistical analysis

RevMan 5.3.5 software (Cochrane Collaboration, London, United Kingdom) was used to synthesize the data. Continuous variables were expressed as mean difference (MD) with a 95% confidence interval (CI), and dichotomous variables as odds ratio (OR) with a 95% CI. Statistical significance was denoted by a two-tailed *p* < 0.05. Chi-square and I^2^ tests were used to test the heterogeneity among trials. Moreover, the fail-safe number was used to estimate the extent of publication bias (Cheng et al., 2016).

### 2.2 Network pharmacology

#### 2.2.1 Data collection

The ingredients of Cordyceps were collected from TCMSP (http://tcmspw.com/tcmsp.php), which is a system pharmacology platform designed for studying TCM comprehensively. Our condition was set with compounds OB ≥ 30%, DL ≥ 0.18, while some ingredients that did not meet the criteria, but had significant pharmacological activity were also selected for the next step. We retrieved the targets of tachyarrhythmia (atrial flutter, atrial fibrillation, premature ventricular beat, ventricular tachycardia, ventricular flutter, ventricular fibrillation, junctional escape and escape rhythm, nonparoxysmal junctional tachycardia, premature contraction of the atrioventricular junction, and sinus tachyarrhythmia), as well as the targets of bradyarrhythmia (sinus bradycardia, sinus arrest, sinoatrial block, atrioventricular block, intraventricular block, sick sinus syndrome) from GeneCards (https://www.genecards.org/), which are online servers for target identification. After gathering all the data, we took the intersections of Cordyceps and disease targets using the online drawing tool called Interactive Venn (http://www.interactivenn.net/).

#### 2.2.2 Protein–protein interaction network construction

The PPI network of the genes was constructed using STRING (https://string-db.org/cgi/input.pl). The protein interaction network was downloaded from its “string_interactions.tsv” format file. For the list of names, we used common targets of Cordyceps with tachyarrhythmia and bradyarrhythmia. After finishing construction, we hid disconnected nodes in the network. Cytoscape 3.9 was used to build the protein-protein interaction network construction.

#### 2.2.3 Functional and pathway enrichment analysis

For the biological and functional annotation of the genes within each herb-disease-target network and PPI, gene ontology (GO) analysis and Kyoto Encyclopedia of Genes and Genomes (KEGG) pathway enrichment analysis were performed using Metascape (http://www.metascape.org/). The function activities and the pathways were ranked by their nominal *p* values with a cut-off of 0.05.

### 2.3 Molecular docking

**FIGURE 1:**
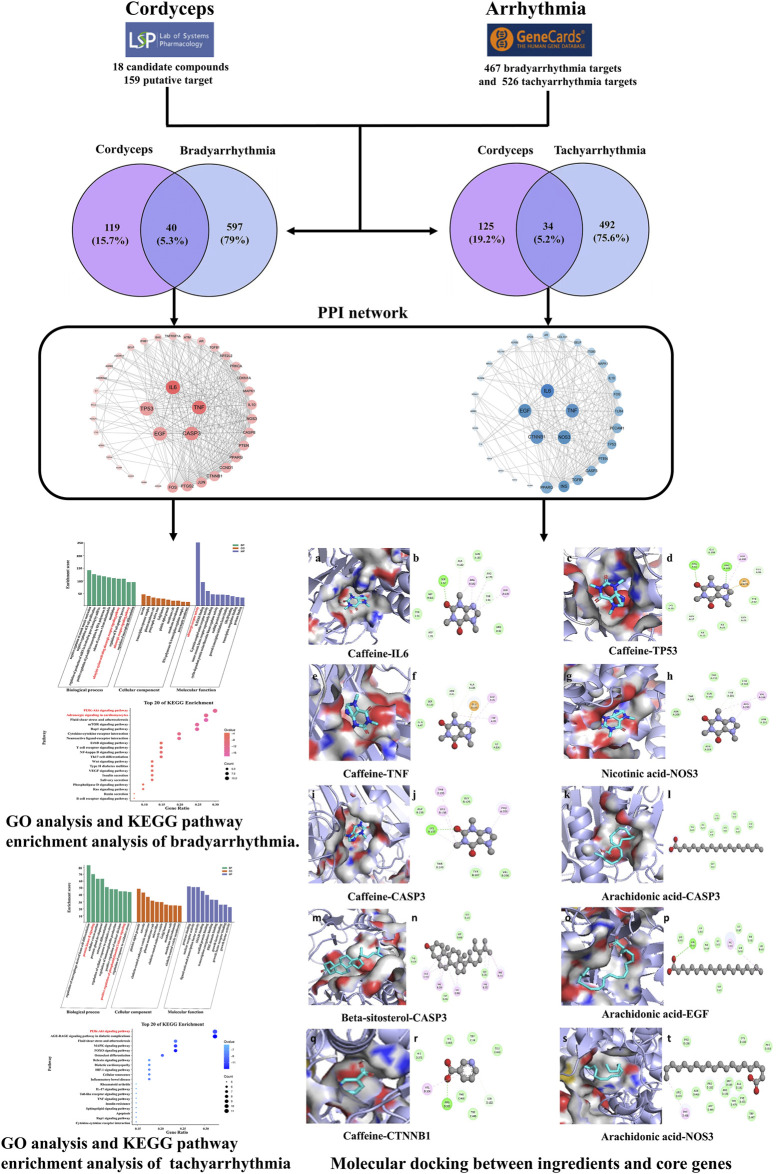
Flow chart investigating Cordyceps in the treatment of arrhythmia.

The 2D structural information was downloaded from the PubChem database (https://pubchem.ncbi.nlm.nih.gov/), and its mechanical structure was optimized by Chem3D software. The Uniprot database (https://www.uniprot.org/) was searched, and the 3D structures regulating the candidate target proteins in the PDB database were downloaded (http://www.rcsb.org/). Autodock tools 1.5.6 (http://autodock.Scripps.Edu/resources/tools) were used for water removal and hydrogenation, for calculating Gasteiger charge, and storing targets and bioactive components as a “pdbqt” format file. The binding conformations were visualized by PyMOL 2.4.0 and Discovery Studio 2019. A flowchart of the network pharmacology study is given in Figure 1.

## 3 Results

### 3.1 Pooled analysis

#### 3.1.1 Study characteristics

In the end, 19 RCTs were included, all of which were published in Chinese. Six trials reported on bradyarrhythmia; the other 13 on tachyarrhythmia. The characteristics of the eligible 19 RCTs are listed in Table 1. The sample sizes ranged from 20 to 180 participants. The 19 RCTs enrolled 1,805 participants, of whom 918 were treated with Ningxinbao capsule plus routine drugs, and, as the control, 887 were treated with only routine drugs. The dosage of Ningxinbao capsule was 0.25 g thrice daily in one trial, and 0.5 g thrice daily in the others. Treatment duration varied from 2 weeks to 1.5 years, and 16 trials used the efficacy rate as the primary outcome. Fourteen trials reported drug-related adverse reactions, including skin allergy, nausea, vomiting, abdominal discomfort, etc. All 19 trials were parallel group RCTs, the Jadad score was 4 in two trials, and 3 in the others.

**TABLE 1:** Meta-analysis of arrhythmia patients’ treatment with Cordyceps.

Category	*n*	Participants, n (cases/controls)	Heterogeneity	RR (95%CI)	Z-test
			P_h_ I^2^ (%)		
Effective rate					
Bradyarrhythmia	5	236/225	0.18 37	1.24 (1.15–1.35)	z = 5.42; *P*_*z*_ <0.00001
Ventricular arrhythmia	5	259/257	0.08 51	1.27 (1.17–1.39)	z = 5.51; *P*_*z*_ <0.00001
Atrial arrhythmia	4	145/145	0.36 7	1.40 (1.20–1.63)	z = 4.24; *P*_*z*_ <0.0001
Multiple types of arrhythmias	4	182/176	<0.0001 88	1.34 (1.19–1.51)	z = 4.75; *P*_*z*_ <0.00001
Incidence of disease					
Ventricular premature beat	1	40/41	NA NA	0.34 (0.10–1.17)	z = 1.71; *P*_*z*_ = 0.09
Ventricular tachycardia	1	40/41	NA NA	1.02 (0.22–4.78)	z = 0.03; *P*_*z*_ = 0.97
Ventricular flutter	1	40/41	NA NA	0.51 (0.05–5.43)	z = 0.56; *P*_*z*_ = 0.58
Ventricular fibrillation	1	40/41	NA NA	0.13 (0.02–0.98)	z = 1.98; *P*_*z*_ = 0.05

*P*_*z*_<0.05 shows a significant association. CI, confidence interval; NA, not available; RR, relative risk; Ph, *p*-values.

#### 3.1.2 Clinical endpoints

##### 3.1.2.1 Bradyarrhythmia

A total of six trials (Zhang & Fan, 2009; Liu et al., 2014; Zeng, 2015; Zhong, 2016; Wang, 2017; Chen, 2018) reported on bradyarrhythmia, enrolling 641 participants, of whom 332 were treated with Ningxinbao capsule plus routine drugs, and, as the control, 309 were treated with only routine drugs. All the participants received routine treatments, including treating the primary disease, reducing blood pressure, decreasing myocardial oxygen consumption, improving myocardial blood supply, strengthening myocardial nutrition, and lipid regulation. The Jadad score was 4 in one trial (Zhong, 2016) and 3 in the others (Zhang & Fan, 2009; Liu et al., 2014; Zeng, 2015; Wang, 2017; Chen, 2018). Five trials used the effective rate as the primary outcome (Liu et al., 2014; Zeng, 2015; Zhong, 2016; Wang, 2017; Chen, 2018). Analyses from five eligible trials with 461 patients (236 in the Ningxinbao capsule groups and 225 in the control groups) exhibited a higher effectiveness rate in patients treated with Ningxinbao capsule plus routine therapy, compared with routine treatment alone (RR = 1.24; 95% CI, 1.15 to 1.35; z = 5.42; P_z_ < 0.00001; I^2^ = 37%) (Table 1). Among the studies that provided a comparison, there was no significant difference in heart rate between the groups with and without Ningxinbao capsule after treatment. Pooled data from two trials (Zhang & Fan, 2009; Zeng, 2015) enrolling 272 patients (142 in Ningxinbao groups and 130 in control groups) indicated that the patients in the Ningxinbao groups had a significant increase in average heart rate (SMD = 1.10; 95% CI, 0.85 to 1.36; z = 8.44; P_z_ < 0.00001; I^2^ = 0%) and minimal heart rate (SMD = 0.99; 95% CI, -0.49 to 2.46; z = 1.31; P_z_ = 0.19; I^2^ = 97%), compared with the control groups (Table 2).

**TABLE 2:** Meta-analysis of arrhythmia patients’ treatment with Cordyceps.

Category	n	Participants, n (cases/controls)	Heterogeneity	SMD (95%CI)	Z-test
			P_h_ I^2^ (%)		
Average heart rate					
Bradyarrhythmia	2	142/130	0.92 0	1.10 (0.85–1.36)	z = 8.44; *P*_*z*_<0.00001
Tachyarrhythmia	2	110/108	<0.00001 97	−1.14 (−2.83 to 0.56)	z = 1.32; *P*_*z*_ = 0.19
Minimal heart rate					
Bradyarrhythmia	2	142/130	<0.00001 97	0.99 (−0.49–2.46)	z = 1.31; *P*_*z*_ = 0.19
Inflammation index					
Hs-CRP	1	66/66	NA NA	2.86 (2.37–3.35)	z = 11.46; *P*_*z*_<0.00001
IL6	1	66/66	NA NA	2.29 (1.84–2.73)	z = 10.15; *P*_*z*_<0.00001
TNF	1	66/66	NA NA	2.13 (1.70–2.56)	z = 9.72; *P*_*z*_<0.00001

*P*_*z*_<0.05 shows a significant association. CI, confidence interval; NA, not available; SMD, standardized mean difference; Ph, *p*-values.

##### 3.1.2.2 Tachyarrhythmia

A total of 13 trials (Xu & Zheng, 1992; Wang et al., 2002; Wang et al., 2009; Zhao, 2012; Wu, 2013; Tao et al., 2014; Li et al., 2016; Wang, 2016; Liu & Chen, 2018; Chen & Zhang, 2019; Cai et al., 2020; Wang, 2020; Cao et al., 2021) reported on tachyarrhythmia. enrolling 1,164 participants, of whom 586 were treated with Ningxinbao capsule plus routine drugs, and, as the control, 578 with only routine drugs. All the participants undertook routine treatment for both the primary disease and the relief of symptoms. Eleven trials (Xu & Zheng, 1992; Wang et al., 2002; Wang et al., 2009; Zhao, 2012; Wu, 2013; Li et al., 2016; Liu & Chen, 2018; Chen & Zhang, 2019; Cai et al., 2020; Wang, 2020; Cao et al., 2021) reported total effectiveness rate as an outcome measure, and our study demonstrates that the total effectiveness rate of the experimental group was higher than the control group, with the difference being statistically significant with obvious heterogeneity. Among the different types of tachyarrhythmia, including three subgroups, results were as follows: ventricular arrhythmia (RR = 1.27; 95% CI, 1.17 to 1.39; z = 5.51; P_z_ < 0.00001), atrial arrhythmia (RR = 1.40; 95% CI, 1.20 to 1.63; z = 4.24; P_z_ < 0.0001), and multiple types of arrhythmias (RR = 1.34; 95% CI, 1.19 to 1.51; z = 4.75; P_z_ < 0.00001) (Table 1).

Two trials (Wang et al., 2009; Cai et al., 2020) of 218 patients (110 in Ningxinbao groups and 108 in control groups), indicated that the patients in the Ningxinbao groups had a significant increase in average heart rate (SMD = −1.14; 95% CI, −2.83 to 0.56; z = 1.32; P_z_ = 0.19; I^2^ = 0%) (Table 2).

One trial (Tao et al., 2014) reported the incidence of ventricular tachyarrhythmia, ventricular premature beat, ventricular flutter, and ventricular fibrillation during the Ningxinbao capsule treatment and follow-up. The result showed that the groups treated with Ningxinbao capsule had a greater reduction in the occurrence of ventricular premature beat (RR = 0.34; 95% CI, 0.10 to 1.17; z = 1.75; P_z_ = 0.09), ventricular tachyarrhythmia (RR = 1.02; 95% CI, 0.22 to 4.78; z = 0.03; P_z_ = 0.97), ventricular flutter (RR = 0.51; 95% CI, 0.05 to 5.43; z = 0.56; P_z_ = 0.58), and ventricular fibrillation (RR = 0.13; 95% CI, 0.02 to 0.98; z = 1.98; P_z_ = 0.05) (Table 1). One trial (Xu & Zheng, 1992) reported on the improvement of symptoms, and here the Ningxinbao capsule had a mild effect on relieving palpitation and chest distress caused by premature ventricular contraction (PVC). One trial (Cao et al., 2021) reported on a comparison of serum inflammatory factors between two groups that showed after 2 months of treatment that the levels of hs-CRP (SMD = 2.86; 2.37 to 3.35; z = 11.46; P_z_ < 0.00001), IL6 (SMD = 2.29; 1.84 to 2.73; z = 10.15; P_z_< 0.00001), and TNF (SMD = 2.13; 1.70 to 2.56; z = 9.72; P_z_ <.00001) in the Ningxinbao capsule group were better than those in the control group (Table 2).

#### 3.1.3 Adverse reactions

##### 3.1.3.1 Bradyarrhythmia

Six trials on bradyarrhythmia (Zhang & Fan, 2009; Liu et al., 2014; Zeng, 2015; Zhong, 2016; Wang, 2017; Chen, 2018) reported drug-related adverse reactions. Three trials reported drug-related adverse events quantitatively (Zhong, 2016; Wang, 2017; Chen, 2018), and the results of these three trials showed that the incidence of adverse events in the Ningxinbao capsule group was 0%, 2.22%, and 0%, respectively, and the incidence of adverse events in the control group was 19.6%, 7.8%, and 17.9%, respectively. The incidence of adverse digestive system events in the Ningxinbao capsule group was significantly lower than in the control group.

##### 3.1.3.2 Tachyarrhythmia

Eight trials reported drug-related adverse reactions quantitatively (Wang et al., 2002; Wu, 2013; Tao et al., 2014; Li et al., 2016; Wang, 2016; Chen & Zhang, 2019; Chen & Zhang, 2019; Cai et al., 2020; Cao et al., 2021), and a distinct difference was observed between the groups with and without the Ningxinbao capsule treatment in terms of adverse effect, which proved that the groups that included the Ningxinbao capsule had less adverse effects on patients’ cardiovascular and digestive systems.

### 3.2 Network pharmacology

#### 3.2.1 Data collection

**FIGURE 2:**
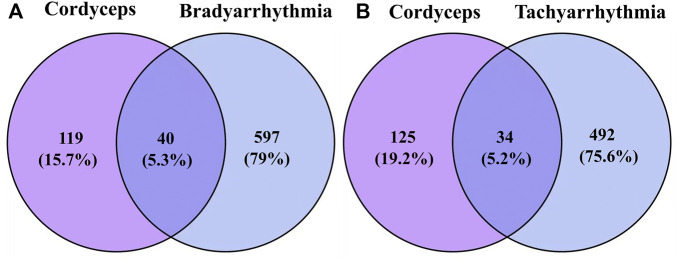
Venn diagram. **(A)** Purple stands for 159 targets of Cordyceps from TCMSP, blue stands for 526 targets of tachyarrhythmia, 40 in the central overlapping section are intersecting Cordyceps and bradyarrhythmia targets. **(B)** Purple stands for 159 targets of Cordyceps from TCMSP, blue stands for 647 targets of tachyarrhythmia, 34 in the central overlapping section are intersecting Cordyceps and tachyarrhythmia targets.

Cordyceps, tachyarrhythmia, and bradyarrhythmia were selected for network pharmacology analysis. In total, 18 main ingredients from Cordyceps were described in the TCMSP database, and 159 related targets were collected. The main ingredients of Cordyceps are listed in Supplementary Table S2. We retrieved 526 and 467 genes from the GeneCards database for tachyarrhythmia and bradyarrhythmia, respectively. Cordyceps shared 34 common targets with bradyarrhythmia and 40 common targets with tachyarrhythmia. The intersections of Cordyceps and disease targets are displayed in Figure 2.

#### 3.2.2 Protein–protein interaction network construction

**FIGURE 3:**
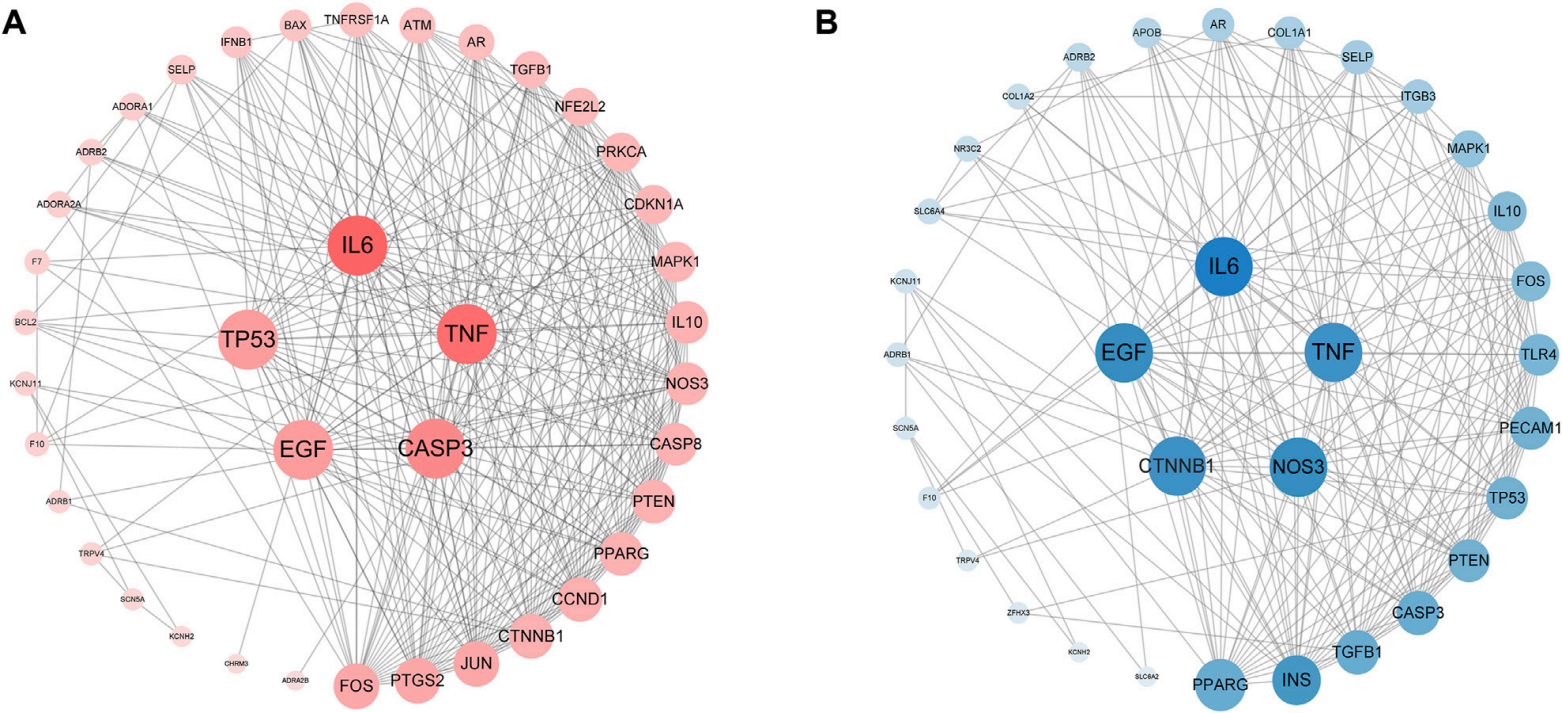
Protein–protein interaction network. **(A)** The pink nodes represent the targets of bradyarrhythmia; the PPI network structure of bradyarrhythmia has 40 nodes and 321 edges. **(B)** The blue nodes represent the targets of tachyarrhythmia; the PPI network structure of tachyarrhythmia has 33 nodes and 207 edges. The sizes of the nodes of the two images are illustrated from large to small in descending order of degree values.

In order to speculate on the role of targets in the disease, overlapping bradyarrhythmia and tachyarrhythmia targets were submitted to a string platform to establish the PPI network. Targets and interacting edges were sorted in descending order by degree, and arranged in a concentric circle according to a degree, as shown in Figure 3. After hiding disconnected nodes in the network, there were 40 targets with bradyarrhythmia and 33 with tachyarrhythmia, with the targets in the innermost circle predicted to be important.

#### 3.2.3 Functional and pathway enrichment analysis

GO functional analysis and KEGG pathway enrichment analysis were conducted to elucidate the biological effects on gene functions and signaling pathways of the related targets of Cordyceps in the treatment of arrhythmia. The top 10 GO items and top 20 KEGG pathways were selected based on the *p* values.

**FIGURE 4:**
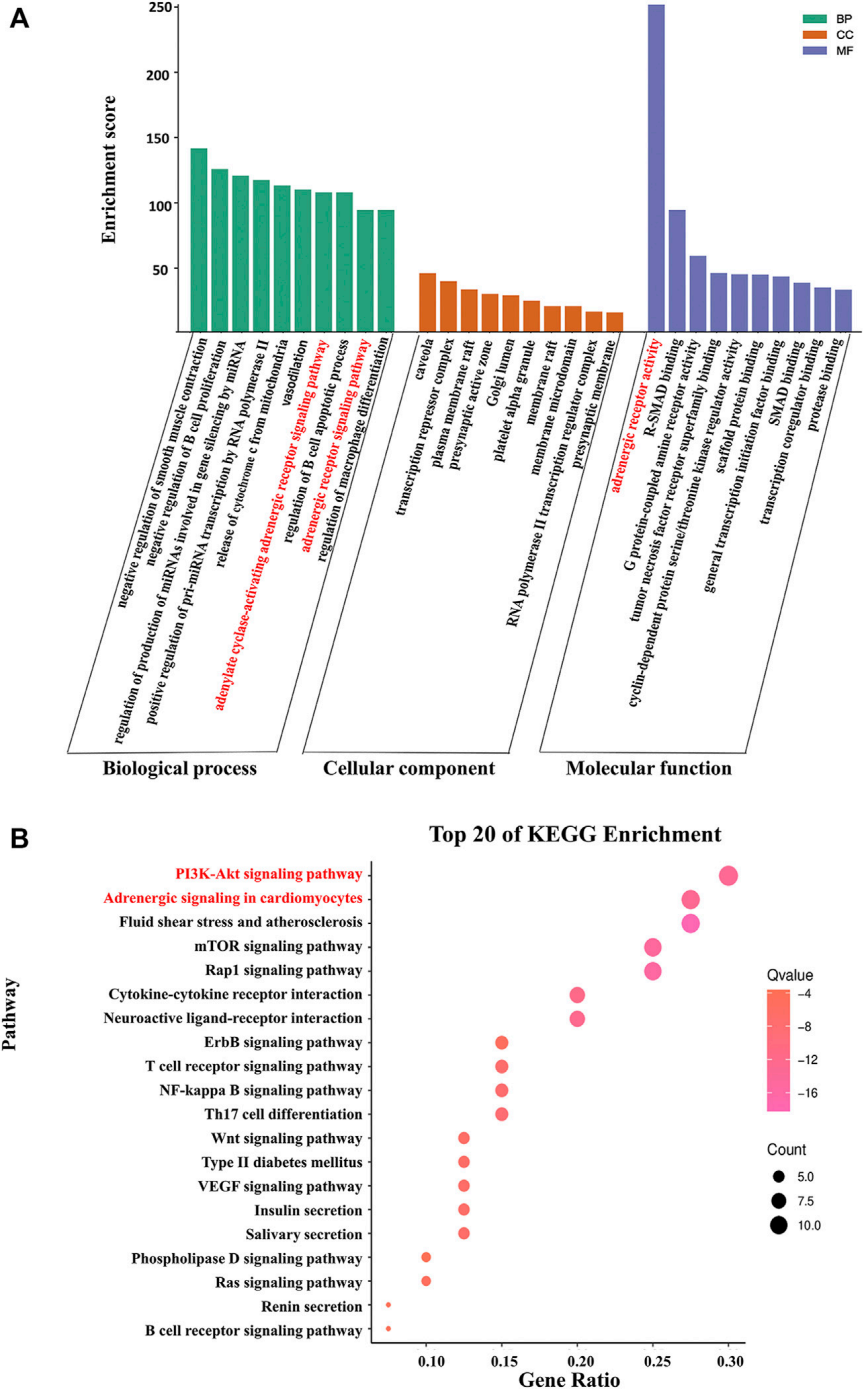
KEGG pathway enrichment analysis and GO functional enrichment analysis of Cordyceps in treating bradyarrhythmia. **(A)** GO functional analysis. The top 10 items of each part are shown. **(B)** KEGG pathway enrichment analysis. The sizes of the bubbles are illustrated from large to small in descending order of the number of potential targets involved in the pathway.

For bradyarrhythmia, the biological processes were mainly concentrated in negative regulation of smooth muscle contraction, negative regulation of B cell proliferation, regulation of production of miRNAs involved in gene silencing by miRNA, positive regulation of pri-miRNA transcription by RNA polymerase II, the release of cytochrome *c* from mitochondria, vasodilation, the adenylate cyclase-activating adrenergic receptor signaling pathway, regulation of the B cell apoptotic process, the adrenergic receptor signaling pathway, and regulation of macrophage differentiation. The cellular components were mainly related to caveolae, the transcription repressor complex, the plasma membrane raft, and the presynaptic active zone. The molecular function was mainly enriched in adrenergic receptor activity, R-SMAD binding, and G protein-coupled amine receptor activity. KEGG pathway results showed that the top 20 pathways were mainly enriched in the PI3K-Akt signaling pathway, adrenergic signaling in cardiomyocytes, fluid shear stress and atherosclerosis, the mTOR signaling pathway, and the Rap1 signaling pathway (Figure 4).

**FIGURE 5:**
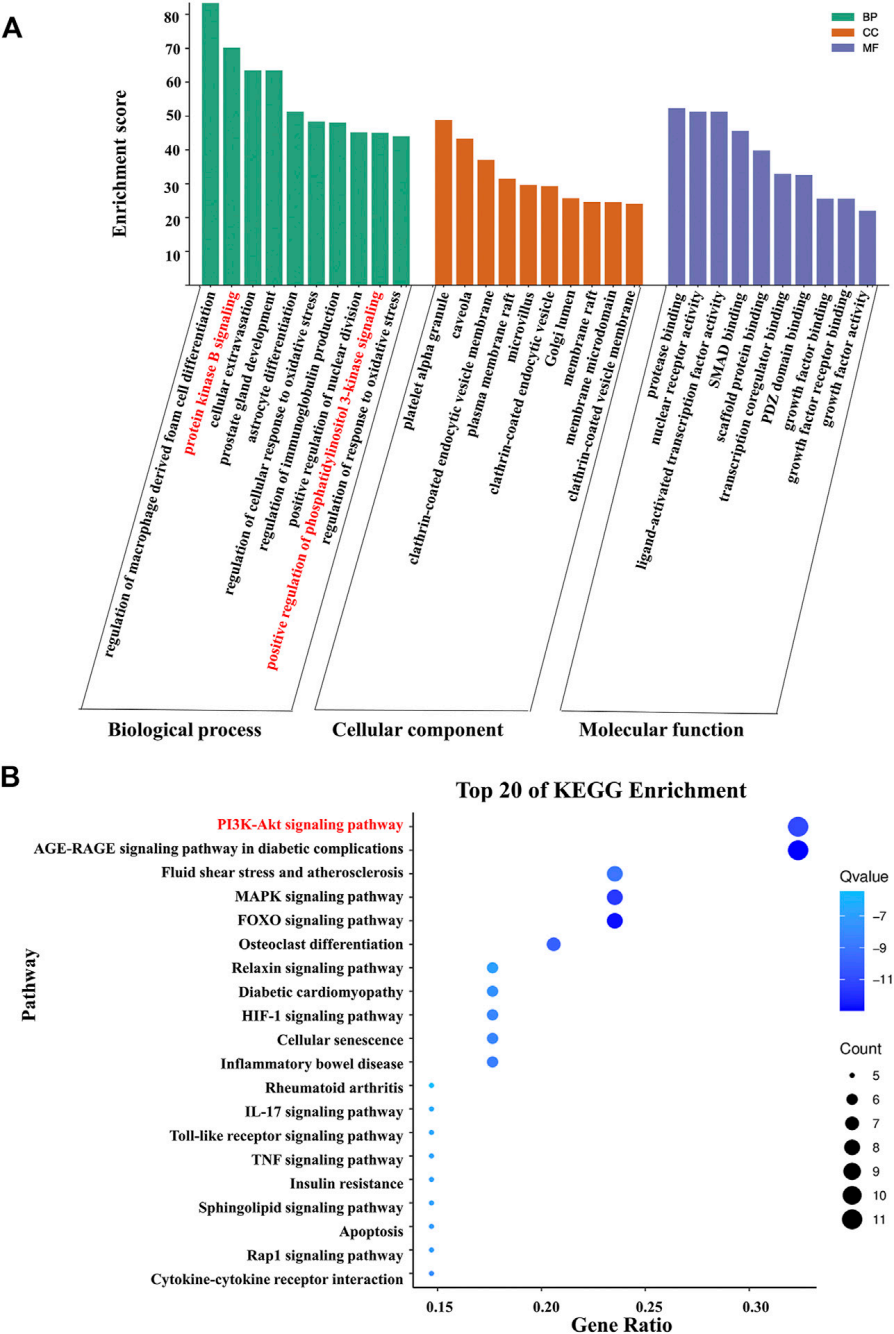
KEGG pathway enrichment analysis and GO functional enrichment analysis of Cordyceps in treating tachyarrhythmia. **(A)** GO functional analysis. The top 10 items of each part are shown. **(B)** KEGG pathway enrichment analysis. The sizes of the bubbles are illustrated from large to small in descending order of the number of potential targets involved in the pathway.

For tachyarrhythmia, the biological processes were mainly concentrated in the regulation of macrophage-derived foam cell differentiation, protein kinase B signaling, cellular extravasation, prostate gland development, astrocyte differentiation, regulation of cellular response to oxidative stress, regulation of immunoglobulin production, positive regulation of nuclear division, and positive regulation of phosphatidylinositol 3-kinase signaling. In terms of cellular components, the top 10 items were mainly related to platelet alpha granules, caveolae, the clathrin-coated endocytic vesicle membrane, and the plasma membrane raft. The molecular function was mainly enriched in protease binding, nuclear receptor activity, ligand-activated transcription factor activity, and SMAD binding. The KEGG pathway analysis revealed that common genes were mainly enriched in the PI3K-Akt signaling pathway, the AGE-RAGE signaling pathway in diabetic complications, fluid shear stress and atherosclerosis, the MAPK signaling pathway, and the FOXO signaling pathway (Figure 5).

### 3.3 Molecular docking

**FIGURE 6:**
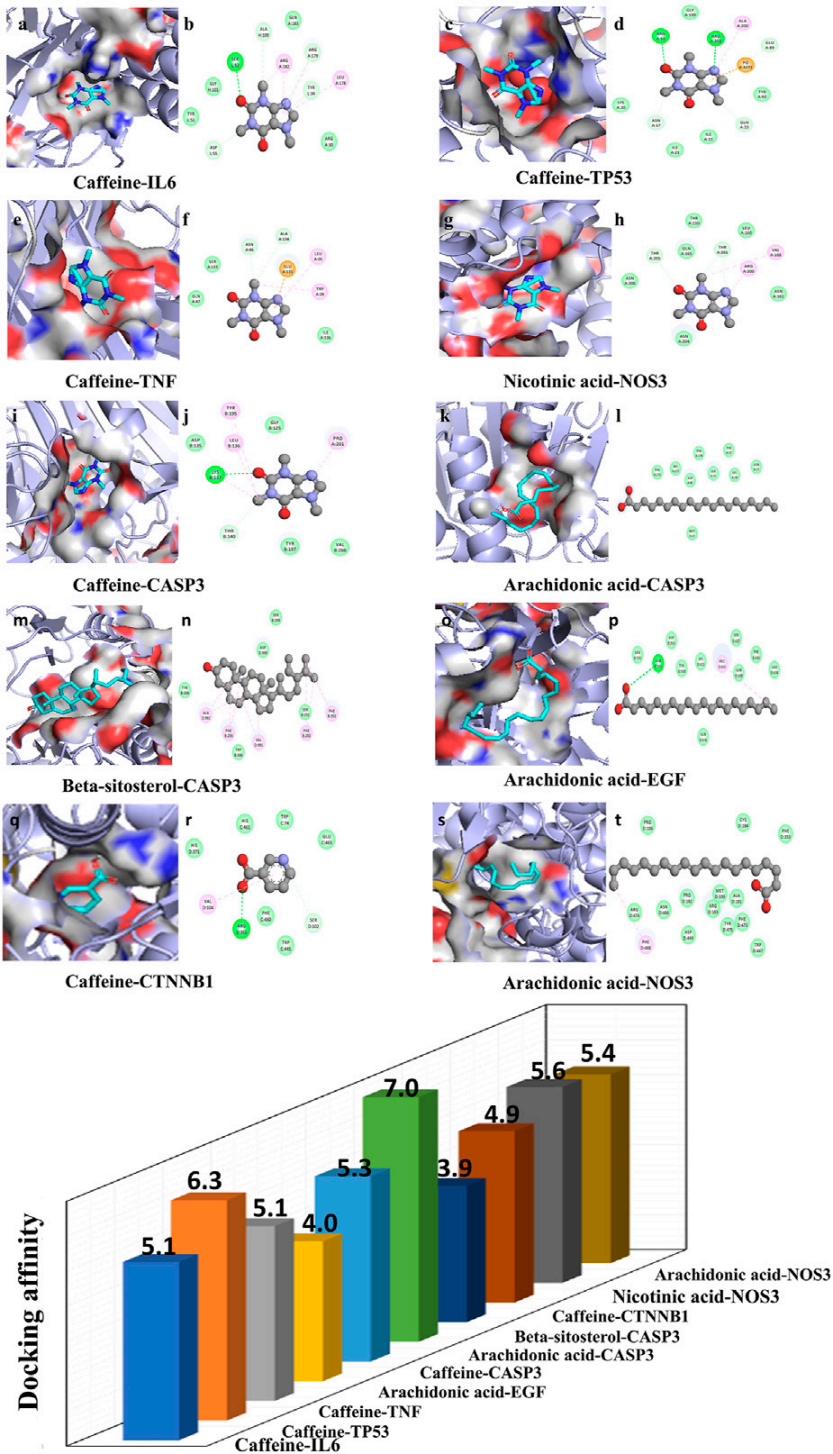
Molecular docking diagram. **(A)** Ten conformations of molecular docking simulation. Diagrams (3D) indicate that the molecular model of the compound is in the binding pocket of the protein. The compound is shown as a stick model with orange coloring. The amino acid residues surrounding are represented by surface style **(A,C,E,G,I,K,M,O,Q,S)**. Diagrams (2D) show the interactions between the compound and surrounding residues **(B,D,F,H,J,L,N,P,R,T)**. **(B)** The 3D column diagram shows the affinity of 10 conformations. *X*-axis: bioactive component, *Y*-axis: target names, *Z*-axis: docking affinity (absolute value of the binding energy). Taking the caffeine-IL6 docking, for example, the small molecule ligand caffeine potentially fits into the interface pocket formed by the interaction of amino acid residues in protein (Figure 6A). As shown in Figure 6 **(B)**, a hydrogen bond was formed with caffeine SER52 near the active site of IL6. The other essential residues (ASP55, TUR51, GLY101, ALA100, GLN183, ARG182, ARG179, TYR34, LEU178 and ARG30) through van der Waal’s forces, carbon hydrogen bond, pi-donor hydrogen bond, pi–pi T-shaped, alkyl, and pi–alkyl. These forms of hydrogen bonds and interactions contribute to the stability of the binding of small molecules to the active sites of proteins.

To confirm the valid bonding effects between ingredients of Cordyceps and key targets, molecular docking was performed by Autodock. The modes of 11 binding complexes are displayed in Figure 6, including caffeine-TNF docking (−5 kcal/mol), caffeine-IL6 docking (−5.1 kcal/mol), caffeine-TP53 docking (−6.3 kcal/mol), arachidonic acid-EGF docking (−4.0 kcal/mol), arachidonic acid-CASP3 docking (−3.9 kcal/mol), beta-sitosterol-CASP3 docking (−7.0 kcal/mol), caffeine-CASP3 docking (−5.3 kcal/mol), caffeine-CTNNB1(−4.9 kcal/mol), arachidonic acid-NOS3(−5.6 kcal/mol), and nicotinic acid-NOS3(−5.4 kcal/mol). The docking results are shown as molecular surface representations, which can effectively reflect the topical details of binding sites. The binding sites are shown in different colors on the protein surface, and hydrogen bonds are shown as dotted lines (Figure 6A). The absolute values of binding affinities (kcal/mol) of all docking patterns indicate a stable combination, and the details of the binding affinities are shown in Figure 6B.

## 4 Discussion

**FIGURE 7:**
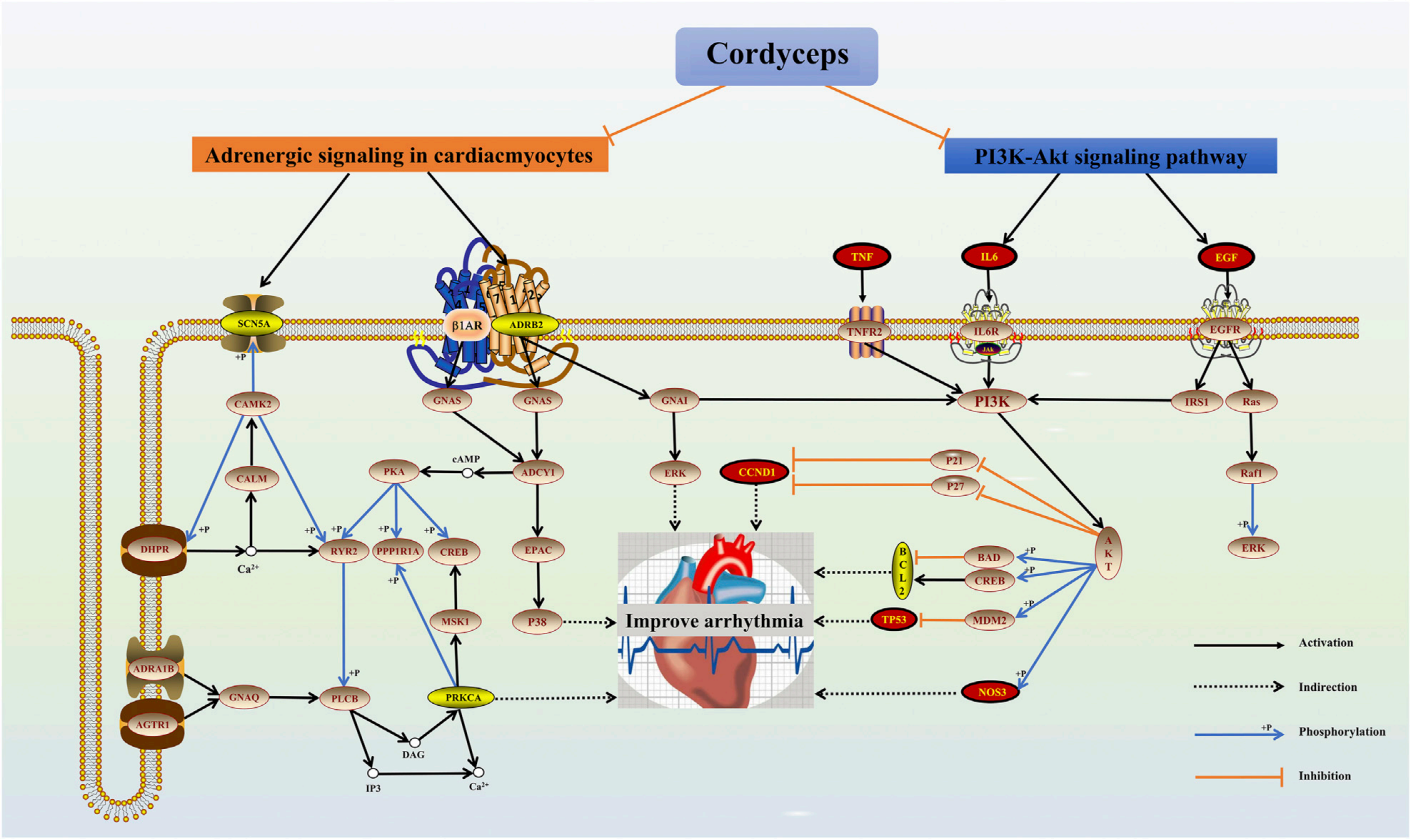
Mechanisms underlying the action of Cordyceps, a traditional Chinese medicine in cardiac function improvement. The red nodes represent the hub genes, the yellow nodes represent common genes, and the other nodes are the genes of the pathway.

In this study, we investigated the efficacy, safety, and potential protective mechanism of Cordyceps in the treatment of arrhythmia. We found that Cordyceps could improve sinus node and atrioventricular conduction in patients with arrhythmia, without obvious adverse reactions. Its possible mechanism of protection is the modulation of adrenergic signaling in cardiomyocytes and the PI3K-Akt signaling pathway (Figure 7).

According to previous studies, the petroleum ether extract of Cordyceps improves the arrhythmia caused by aconitine, prolongs the induction time of arrhythmia, and reduces the duration and severity of arrhythmia in rats. The extract of Cordyceps also has an antagonistic effect on arrhythmia induced by barium chloride. The mechanism of its anti-arrhythmic action might involve the regulation of the transport of ion channels in the cell membrane (Mei et al., 1989). In our meta-analysis, Cordyceps exerts dual regulatory effects on arrhythmias. The underlying mechanisms of Cordyceps in the treatment of bradycardia are as follows: 1) Cordyceps not only increases the sinus heart rate but also improves the electrical activity of the sinus node and atrioventricular conduction. 2) Cordyceps could increase the blood flow of the heart and brain, improve microcirculation, and stabilize blood pressure. 3) Cordyceps might improve the activity of fibrinolytic enzymes, promote fibrinolysis, and reduce blood viscosity. 4) Cordyceps might increase the activity of circulating superoxide dismutase and inhibit the production of lipid peroxides and free radicals (Zeng, 2015; Das et al., 2020).

Cordyceps contains a variety of chemical components, such as sterols amino acids, polysaccharides, organic acids, nucleosides, peptides, and trace elements. Caffeine plays an important role in preventing and treating arrhythmia. Previous studies have found that taking a certain amount of caffeine before aerobic exercise can delay the recovery of autonomic control of heart rate in young adults (Gonzaga et al., 2017). Low doses of caffeine can reduce heart rate during submaximal cycle ergometry (Flueck et al., 2016), while a high dose of caffeine (a dose of 150°mg) increases the heart rate of healthy people (Vukovich et al., 2005). The effect of caffeine on heart rate may be one of the reasons for the bidirectional regulation of heart rate by Cordyceps. It has been reported that beta-sitosterol can reduce blood lipids and prevent a variety of cardiovascular diseases. Beta-sitosterol can inhibit the arrhythmia induced by aconitine, ouabain, and myocardial ischemia (Zhang et al., 2013). Cinnamon is a plant with strong antioxidant activity. Due to its antioxidant properties, the ethanol extract of cinnamon bark protects the heart from ischemia-reperfusion injury (Sedighi et al., 2018). Arachidonic, linoleic, stearic, and oleic acids are the fatty acids in the human body that can reduce oxidative stress and hence help prevent cardiovascular disease (Sakai et al., 2017). Fatty acids and arachidonic acid prevent arrhythmias by modulating ion channel homeostasis, while intravenous application of arachidonic acid and linoleic acid-induced strong antiarrhythmic effects in three experimental arrhythmia models (Forster et al., 1976; Xiao et al., 2004). In order to better explore the mechanism of action of Cordyceps, we collected the common targets of Cordyceps, bradyarrhythmia, and tachyarrhythmia through network pharmacology constructed a PPI network, and performed enrichment analysis. Interestingly, two of the top 10 biological process items for bradyarrhythmia are associated with the adrenergic receptor and have molecular functional references to that receptor. According to KEGG results, which mainly enriched the PI3K-Akt signaling pathway and adrenergic signaling in cardiomyocytes, it is possible that the PI3K-Akt signaling pathway and adrenergic signaling in cardiomyocytes act together to regulate bradyarrhythmia. Two of the biological processes for tachyarrhythmia mentioned were positive regulation of protein kinase B signaling and phosphatidylinositol 3-kinase signaling. The results of the KEGG enrichment analysis suggest that Cordyceps may play a role in the treatment of tachyarrhythmia through PI3K-Akt signaling. Subsequently, through molecular docking, the results showed that Cordyceps has good docking activity with the core gene hints of bradyarrhythmia and tachyarrhythmia targets.

According to the analysis of the PPI network, IL6, TNF, TP53, CASP3, CTNNB1, EGF, and NOS3 may be the key targets of Cordyceps in the treatment of arrhythmia. Previous studies have found that acute administration of IL6 to isolated rat hearts could recapitulate part of the Ca^2+^ handling phenotype. In addition, intraperitoneal injection of IL-6 in rats increased susceptibility to atrial fibrillation (Liao et al., 2021). TNF⁃α is a proinflammatory cytokine, which increases in several cardiac diseases. TNF-α rapidly increased spontaneous calcium release activity in mouse atrial cells via the mitochondrial reactive oxygen species pathway and promoted the possibility of arrhythmia-triggered activity (Zuo et al., 2019). There is growing evidence that IL-6 and TNF have significant inhibitory effects on myocardial metabolic processes in both ventricles, which can worsen the course of cardiovascular disease and increase the risk of tachyarrhythmia (Tverskaya et al., 2017). It has been experimentally confirmed that the mechanism of trimethyltin chloride induced bradyarrhythmia may indicate a close correlation between Na^+^/K^+^-ATPase activity and CASP3 protein expression (Liu et al., 2020). Cardiac tissue-specific plakoglobin and CTNNB1 are essential for the maintenance of cardiac conduction, while cardiac tissue-specific plakoglobin and rent series proteins are required to anchor the n-calcium mucoprotein/serial protein adhesion complex on the cytoskeleton. Loss of this connection leads to disruption of cardiac intercalated disc structure and the failure of the fatal cardiac arrhythmia (Swope et al., 2012). Previous studies have observed an increased incidence of arrhythmias in NOS3^−/−^mice. NOS3 may prevent arrhythmias by modulating Ca^2+^ transient amplitude (Wang et al., 2008). TP53 is a transcription regulator and a significant tumor suppressor. Notably, TGF-β1 and TP53 in transplanted left ventricular tissue suggest a direct link between these genes and ventricular arrhythmias (Huizar et al., 2019; Haywood et al., 2020). Experimental results suggest that by interfering with beta-adrenergic receptor signaling, EGF protects the heart from the deleterious effects of adrenaline (Lorita et al., 2002).

The main mechanism of arrhythmia is a change in the physiological characteristics of the Na^+^, K^+,^ and Ca^2+^ channels of the myocardial cell membrane, which leads to abnormal autonomic activity, triggering activity, and reentry excitement of the myocardium. Adrenergic receptors, as G protein-coupled receptors, play a key role in cardiac physiology in both health and disease. Previous investigations have indicated that cardiac adrenal receptors are directly involved in the control of intercellular electrical communication, which may be a key factor in maintaining normal intercellular conduction and the cardiac electrical network. This may in turn be related to the formation of arrhythmogenic substrates in some heart diseases (Salameh and Dhein, 2011). Adenine dinucleotide phosphate nicotinic acid is the most potent second messenger for Ca^2+^ release known to date. Stimulation of beta-adrenaline leading to Ca^2+^ release can induce arrhythmias (Nebel et al., 2013). The binding of sympathetic nervous system agonists to adrenergic receptors in cardiac myocytes can increase heart rate and myocardial contractility through cascade signaling pathways and protein kinase A (Papa et al., 2022). Enhanced α1-adrenoceptor stimulation affects intracellular Ca^2+^ signaling, thereby regulating heart rate (Luo et al., 2007). It has been shown in animal studies that α 1-adrenergic receptors can influence heart rate by inducing positive or negative inotropic responses by altering K^+^ and Ca^2+^ currents, intracellular pH, and Ca^2+^ sensitivity of myofilaments (O’connell et al., 2014). Beta-adrenergic receptors induced an acute inhibitory K^+^ response in the hearts of aging guinea pigs, and thus inhibited those receptors in reducing the occurrence of arrhythmias (Zou et al., 2021).

The movement of Ca^2+^ through the cell membrane generates various electrical currents to stimulate action potential, determining the cardiac cycle and cardiac function (Tosaki, 2020). Previous investigations have indicated that allicin may inhibit Ca^2+^ overload-induced apoptosis and the PI3K-mediated GRK2/PLC-γ/IP3R signaling pathway to protect against myocardial injury (Gao et al., 2021). In cardiomyocytes, PI3K-mediated regulation of Cav2.2α1/β2a transport is considered to be a general mechanism for regulating Ca^2+^ entry into excitatory cells (Viard et al., 2004). In addition, the fact that Akt regulates the C-terminal region of the Ca^2+^ channel function CaVβ2 through mediated phosphorylation of CaVβ2 in all excitable cells, including cardiomyocytes, highlights the biological relevance of this mechanism. More importantly, phosphorylation of CaVβ2 is thought to affect calcium influx and myocardial contractility, which is closely associated with the mechanism of arrhythmia (Ghigo et al., 2017). Previous research also indicates that elevated levels of angiotensin II may indirectly inhibit PI3Kα, and ultimately reduce the number of L-type Ca^2+^ channels in cardiac myocytes. These studies confirmed that PI3Kα is the main regulator of Ca^2+^ current regulation in cardiomyocytes (Liang et al., 2010). PI3K is believed to indirectly regulate ptdIns-3,4, 5-P3 in calcium signal transduction. The release of activated Ca^2+^, in addition to ptdIns-3,4, 5-P3, has been identified as part of a signaling pathway used to regulate the cytoplasmic calcium concentration, suggesting that the functional link between the PI3K-Akt pathway and the calcium signaling pathway may be of broad importance (Blair & Marshall, 1997; Scharenberg & Kinet, 1998). The PI3K-Akt pathway is a key pathway for cardiac protection under stress conditions. Mice with reduced cardiac PI3K-Akt activity are very sensitive to atrial fibrillation, and clinical studies have found that surgical specimens from patients with atrial fibrillation showed significantly lower cardiac PI3K-Akt activity than patients with sinus rhythm (Pretorius et al., 2009). The cardioprotective role of this pathway has been further highlighted by studies showing that increased PI3K-Akt activity improves the function of failing hearts in mouse models of heart failure (Mcmullen et al., 2014).

Our study presented a relatively strict illustration of the results. However, there were still some limitations. First, all trials included were published in Chinese and their sample sizes were small. Since all studies included were conducted in China, there might be a geographic bias, which limits generalization to the world. Second, all studies included reported positive results, which might reflect publication bias. Further, strictly designed multicenter RCTs are needed to assess the effectiveness and safety of TCM, especially long-term hard endpoints. Finally, in this study, the molecular mechanisms of Cordyceps were predicted only through network pharmacology, so further experiments are needed to confirm these mechanisms.

Taken together, based on meta-analysis, network pharmacology, and molecular docking technology, the results of our study suggest that Cordyceps is effective in stabilizing heart rates of patients with arrhythmia. It is hypothesized that Cordyceps may play a role in the treatment of cardiac arrhythmias by modulating adrenergic signaling in cardiomyocytes and the PI3K-Akt signaling pathway. However, the effect of Cordyceps on long-term outcomes in these patients remains unknown. It is hoped that our study will provide more references for the treatment of arrhythmia.

## Data Availability

The original contributions presented in the study are included in the article/Supplementary Material, and further inquiries can be directed to the corresponding authors.
