# Supplementary material for: Bidirectional regulatory effects of Cordyceps on arrhythmia: Clinical evaluations and network pharmacology
Source: Front Pharmacol. 2022 Aug 19;13:948173. doi: 10.3389/fphar.2022.948173 (PMC9437265; doi:10.3389/fphar.2022.948173)
Supplement: Supplementary file 1 [file Table1.DOCX]

Supplementary Material

**Table S1. Characteristics of patients, treatment, outcomes and complications that were compared in the included trials.**

| **ID** | **Sample size**  **(treatment/control)** | **Diagnosis** | **Intervention** | **Comparison** | **Treatment duration** | **Outcome measure** |
| --- | --- | --- | --- | --- | --- | --- |
| Chen Y  2018 | 83(45/38) | Bradycardia | NXB capsule 0.5g tid + trimetazidine 0.02g tid  + routine treatment | Trimetazidine 0.02g qd  + routine treatment | 3 months | Total effective rate |
| Wang XY  2017 | 102(51/51) | Bradycardia | NXB capsule 0.5g tid + trimetazidine 0.06g tid  + routine treatment | Trimetazidine 0.06g tid  + routine treatment | 4 weeks | Total effective rate  (specific criterion was unclear) |
| Zhong WZ  2016 | 112(56/56) | Bradycardia | NXB capsule 0.5g tid + trimetazidine 0.02g tid  + routine treatment | Trimetazidine 0.02g qd  + routine treatment | 4 weeks | Total effective rate |
| Zeng JH  2015 | 92(46/46) | Bradycardia | NXB capsule 0.25g tid + trimetazidine 0.02g qd  + routine treatment | Trimetazidine 0.02g qd  + routine treatment | 2 to 3 months | Resting state heart rate, Slowest heart rate,  Average heart rate, |
| Liu JH  2014 | 72(38/34) | Bradycardia | NXB capsule 0.5g tid + trimetazidine 0.02g tid  + routine treatment | Trimetazidine 0.02g tid  + routine treatment | 2 to 3 months | Total effective rate, Dynamic ECG  improvement of symptom |
| Zhang XY  2009 | 180(96/84) | Bradycardia | NXB capsule 0.5g tid + routine treatment | Routine treatment | 80~120 days | Dynamis ECG, Average heart rate, Minimum heart rate per hour |
| Cai JR  2020 | 94(47/47) | Atrial fibrillation | NXB capsule 0.5g tid + amiodarone hydrochloride 0.2g tid for first week, 0.2g bid for second week + 0.2g qd for third week + routine treatment | Amiodarone hydrochloride 0.2g tid for first week + 0.2g bid for second week + 0.2g qd for third week | 12 months | Total effective rate |
| Cao DP  2021 | 132(66/66) | Ventricular arrhythmia, Ventricular tachycardia,  Ventricular fibrillation | NXB capsule 0.5g bid + Propafenone hydrochloride 0.3g tid | Propafenone hydrochloride 0.3g tid | 2 months | Total effective rate |
| Wang XF  2020 | 96(48/48) | Atrial fibrillation | NXB capsule 0.5g tid + Amiodarone hydrochloride 0.2g tid for 15 days + 0.2g bid for 11.5 months | Amiodarone hydrochloride 0.2g tid for 15 days + 0.2g bid for 11.5 months | 12 months | Total effective rate |
| Chen T  2019 | 151(75/76) | Ventricular arrhythmia | NXB capsule 0.5g tid + Propafenone hydrochloride 0.3g tid | Propafenone hydrochloride 0.3g tid | 2 months | Total effective rate |
| Tao YZ  2014 | 81(40/41) | Premature ventricular beat, Ventricular tachycardia, Ventricular flutter, Ventricular fibrillation | NXB capsule 0.5g tid + amiodarone 0.6g tid for 7 days, 0.2~0.4g qd or bid for the 8th to 30th + routine treatment | Amiodarone 0.6g tid for 7 days + 0.2~0.4g qd or bid for the 8th to 30th  + routine treatment | 3 months  follow 6 months | The incident rate of Ventricular tachycardia,  ventricular premature beat,  ventricular flutter,  ventricular fibrillation |
| Wu Y  2013 | 64(34/30) | Premature ventricular beat, Atrial premature beat, Atrial fibrillation | NXB capsule 0.5g tid for two weeks + amiodarone 0.2g tid for one week + 0.1g tid for the second week + routine treatment | Amiodarone 0.2g tid  for two weeks  + routine treatment | 2 weeks | Total effective rate |
| Zhao NH  2012 | 98(50/48) | Premature ventricular beat, Atrial premature beat, Atrial fibrillation, Premature contraction of atrioventricular junction | NXB capsule 0.5g tid + amiodarone 0.3g tid + routine treatment | Amiodarone 0.3g tid  + routine treatment | 2 weeks | Total effective rate |
| Liu YH  2018 | 20(10/10) | Atrial fibrillation | NXB capsule 0.5g tid + Codarone + routine treatment | Codarone  + routine treatment | 1 year | Total effective rate |
| Xu JM  1992 | 64(32/32) | Atrial arrhythmia,  Ventricular arrhythmia | NXB capsule 0.5g tid + routine treatment | Placebo  + routine treatment | 2 weeks | Total effective rate  ECG |
| Wang LD  2015 | 40(20/20) | Atrial fibrillation | NXB capsule 0.5g tid + amiodarone 0.2g tid for first week 0.2g bid for second week, 0.2g qd for third week + routine treatment | Amiodarone 0.2g tid for first week + 0.2g bid for second week + 0.2g qd for third week  + routine treatment | 1 year | Sinus rhythm conversion rate |
| Li XF  2016 | 100(50/50) | Ventricular arrhythmia | NXB capsule 0.5g tid + verapamil 0.12g bid  + routine treatment | Verapamil 0.12g bid  + routine treatment | 4 weeks | Total effective rate  ECG |
| Wang X  2009 | 124（63/61） | Ventricular arrhythmia | NXB capsule 0.5g tid + metoprolol tartrate 12.5mg bid + routine treatment | Metoprolol tartrate 12.5mg bid  + routine treatment | 4 weeks | Average heart rate  Total effective rate |
| Wang SY  2001 | 60(30/30) | Ventricular arrhythmia | NXB capsule 0.5g tid+ Propafenone 0.15g tid + routine treatment | Propafenone 0.15g tid  + routine treatment | 2 weeks | Total effective rate |

Tid, three times a day; bid, twice a day; qd, one time a day; NXB, ningxinbao; ECG, electrocardiogram; NA, not available.
