# Supplementary material for: Bidirectional regulatory effects of Cordyceps on arrhythmia: Clinical evaluations and network pharmacology
Source: Front Pharmacol. 2022 Aug 19;13:948173. doi: 10.3389/fphar.2022.948173 (PMC9437265; doi:10.3389/fphar.2022.948173)
Supplement: Supplementary file 2 [file Table2.DOCX]

Supplementary Material

# Table S2. Main ingredients of Cordyceps.

| **Mol ID** | **Mol Name** | **OB** | **DL** |
| --- | --- | --- | --- |
| MOL000991 | Cinnamaldehyde | 31.99 | 0.02 |
| MOL001308 | Oleic acid | 33.13 | 0.14 |
| MOL001439 | Arachidonic acid | 45.57 | 0.2 |
| MOL001691 | Vitamin c | 13.34 | 0.04 |
| MOL001744 | Uracil | 42.53 | 0.02 |
| MOL001788 | Adenine | 62.81 | 0.03 |
| MOL000421 | Nicotinic acid | 47.65 | 0.02 |
| MOL000059 | Uridine | 10.49 | 0.11 |
| MOL006077 | Thiamine | 19.87 | 0.11 |
| MOL000860 | Stearic acid | 17.83 | 0.14 |
| MOL000069 | Palmitic acid | 19.3 | 0.1 |
| MOL001439 | Arachidonic acid | 45.57 | 0.20 |
| MOL001645 | Linoleyl acetate | 42.10 | 0.20 |
| MOL000358 | Beta-sitosterol | 36.91 | 0.75 |
| MOL001169 | Peroxyergosterol | 44.39 | 0.82 |
| MOL008998 | Cerevisterol | 39.52 | 0.77 |
| MOL008999 | Holesteryl palmitate | 31.05 | 0.45 |
| MOL000953 | CLR | 37.87 | 0.68 |

Mol, molecule; ID, Identity; OB, oral bioavailability; DL, drug likeness; Data sources: TCMSP, Filtering condition: OB≥30.
